# Unheard voices in oral health: the mouth, oral health, and barriers to accessing dental care in disadvantaged communities and Venezuelan migrants in Bogotá

**DOI:** 10.1186/s12939-026-02925-9

**Published:** 2026-07-17

**Authors:** Luis Alberto Sánchez–Alfaro, María Kamila Navarro-Ramírez, Roger Keller Celeste, Richard G. Watt, Michelle Stennett, Alastair Ross, Regina Mutave, Mirai Chatterjee, Starry John D’Cruz, Paulo Sávio Angeiras de Goes, David I. Conway, Carol C. Guarnizo-Herreño

**Affiliations:** 1 Department of Collective Health, School of Dentistry, Universidad Nacional de Colombia, Bogotá, Colombia; 2 Department of Preventive and Social Dentistry, Universidade Federal do Rio Grande do Sul, Porto Alegre, Brazil; 3 Department of Epidemiology and Public Health, University College London, London, WC1E 7HB UK; 4 School of Health, Education, Policing and Sciences, Staffordshire University, Stoke-on-Trent, GB England; 5 University of Nairobi, Nairobi, Kenya; 6 Self Employed Women’s Association (SEWA), Ahmedabad, India; 7 Federal University of Pernambuco, Recife, Brazil; 8 University of Glasgow, Glasgow, UK

**Keywords:** Dental care, Barriers to access of health services, Oral health, Health inequities, Social justice

## Abstract

**Background:**

Research on barriers to oral healthcare in Colombia shows that access is restricted by the uneven geographical distribution of dental providers and high out-of-pocket costs for patients, particularly affecting poor populations, residents of marginalised areas, and people in vulnerable situations. This study aimed to explore the significance given to the mouth, oral health, and the barriers to accessing dental care based on the experiences of people belonging to disadvantaged communities and Venezuelan migrants in Bogotá, Colombia.

**Methods:**

An exploratory qualitative study with a phenomenological approach was conducted. Two data collection tools were used: field diaries and semi-structured interviews. A hermeneutic-phenomenological analysis was performed using a process that included: coding and establishing general units of meaning (initial categorisation), arranging and grouping data (relevant units of meaning); and triangulation of sources and face-to-face validation between researchers.

**Results:**

Three units of meaning were identified: personal confidence and social interaction, barriers to access, and bureaucratic itineraries and dehumanisation. Although the mouth and oral health were identified as fundamental to people’s lives, playing a crucial role in social interactions and self-esteem, people belonging to vulnerable and marginalised communities experience significant barriers that hinder access to oral health care. The main barriers identified were geo-economic and technological.

**Conclusions:**

The bureaucracy of the Colombian health system exacerbates the situation, contributing to the high burden of oral diseases and leaving people without timely care. Public policies must focus on reducing these barriers to ensure equity and effective exercise of the human right to oral health.

**Supplementary Information:**

The online version contains supplementary material available at https://doi.org/10.1186/s12939-026-02925-9.

## Abstract in Spanish

**Antecedentes y objetivo:**

las investigaciones relacionadas con barreras al acceso a la atención odontológica en Colombia muestran que dicho acceso está limitado por la distribución geográfica desigual de los profesionales y los elevados gastos que deben realizar los pacientes, esto afecta especialmente a las poblaciones pobres, residentes de zonas marginadas y personas vulnerables. El objetivo de este estudio fue explorar la significación dada a la boca y a la salud oral, así como las barreras para acceder a la atención odontológica, a partir de las experiencias de personas pertenecientes a comunidades vulnerables y migrantes venezolanos en Bogotá, Colombia.

**Métodos:**

estudio cualitativo exploratorio con enfoque fenomenológico. Se utilizaron dos herramientas de recolección de la información: el diario de campo y la entrevista semiestructurada. Se realizó un análisis de tipo hermeneútico–fenomenológico mediante un proceso de doble vía (deductivo–inductivo) que incluyó: codificación y establecimiento de Unidades de significado general (categorización inicial), disposición y agrupamiento de datos (Unidades de significado relevante); finalmente triangulación de fuentes y validación cara a cara entre investigadores, que permitió la Verificación de Unidades de significado relevante.

**Resultados:**

se proponen tres Unidades de significado relevante para entender la significación de la boca, la salud oral y los procesos de atención odontológica: Confianza personal e interacción social, Barreras de acceso, e Itinerarios burocráticos y deshumanización. Aunque se reconoció que la boca y la salud oral son fundamentales para la vida de las personas, ya que desempeñan un papel crucial en las interacciones sociales y la autoestima, las personas pertenecientes a comunidades vulnerables y marginadas se enfrentan a importantes barreras que dificultan el acceso a la atención odontológica. Las principales barreras identificadas fueron de carácter geo-económico y tecnológico.

**Conclusiones:**

la burocracia del sistema sanitario colombiano agrava la situación, contribuyendo a la elevada carga de morbilidad de las enfermedades orales y dejando a las personas sin atención oportuna. Las políticas públicas deben centrarse en reducir estas barreras para garantizar la equidad y el ejercicio efectivo del derecho humano a la salud oral.

## Background

Oral diseases include a variety of conditions, including dental caries, periodontal disease, and lip and mouth cancer. Although largely preventable, these conditions are among the most common diseases worldwide, affecting nearly 3.7 billion people [1–3]. They significantly affect people’s overall health, well-being, and quality of life [4]. Furthermore, the high cost of treatment has a significant economic impact on family budgets and health systems [5, 6]. Oral diseases are more common among the most marginalised and excluded groups in society. These inequities (avoidable and unfair differences) stem from unequal access to resources, prestige, and power, which manifest in a wide range of social conditions beyond people’s direct control [1, 7, 8].

In many parts of the world, oral health care is neither accessible nor available to the most marginalised groups [9]. This problem is particularly critical in many low- and middle-income countries, where most services are provided to people of high socioeconomic status living in urban areas. The most vulnerable populations —particularly in rural areas, deprived urban settings, ethnic minorities, and undocumented migrants, among others— face significant challenges in accessing quality, culturally competent, and affordable dental care [9, 10].

In Colombia, healthcare services (including dental services) are provided based on different health insurance schemes. Participation in one of these schemes depends on family members’ working status, poverty level, or household resources. The subsidised regime serves individuals without formal employment who qualify as “poor”, with coverage primarily funded by taxation. The contributory regime encompasses those in formal employment or self-employment who can afford monthly contributions. The exceptional regime provides coverage for armed forces members, public-sector educators, and petroleum sector employees. Collectively, these segmented schemes —coupled with persistent financial obstacles— continue to hinder equitable access to healthcare across the country [11–15].

Research on barriers to oral health care in Colombia suggests that access is limited by the unequal geographical distribution of providers and high out-of-pocket costs for patients [13, 15]. Although the Colombian health system’s basic package of health services covers various dental treatments, this is insufficient given structural problems of access, availability, procedures for obtaining authorisations to receive care, discontinuity of government strategies or policies, and weak cooperation between sectors, especially in contexts of high social vulnerability [16–19]. Therefore, it is necessary to adopt an oral health perspective focused on social justice and the recognition of the right to health, in which citizens’ voices are heard and included, particularly those of marginalised communities and people in situations of greater vulnerability.

Taking the above into account, with the aim of contributing from a qualitative perspective, the research underlying this article aimed to give these individuals (marginalised and vulnerable people in Bogotá, Colombia) a ‘voice’ to express their own stories and experiences related to oral health. This study was conducted in the context of the NIHR Global Health Research Group on Oral Health [The CORE programme] [20], and aimed to explore the experiences of people living in socioeconomic disadvantaged areas and Venezuelan migrants in Bogotá (Colombia) with respect to their oral health, perceived barriers to accessing dental care, and experiences with services among those who have used them.

## Materials and methods

A comprehensive exploratory qualitative study was carried out using a phenomenological approach and a hermeneutic perspective. The phenomenological nature of the study lies in its ability to describe experiences as they were lived by those who experienced them in their context, recognizing their perceptions, reasoning, and emotions. The hermeneutic perspective was developed through interpreting participants’ narratives, taking into account their experiences, social context, and ongoing reflection throughout the research process [21–23].

To account for the above, some structural elements were explored, namely: *the lived experience* (what participants experienced and learned) when using oral health services, that is, the objectification of experience of oral health care and attention that allows the production of *common sense* regarding the mouth and oral health; and *the significance* that participants attach to the mouth and oral health in relation to care services and practices within their socioeconomic and cultural context. Together, these elements enabled a deeper understanding of the nature and meaning of experiences related to the mouth, oral health, and barriers to accessing dental care.

Forty-one people participated: 21 Colombians (10 men, 11 women) and 20 Venezuelan migrants (10 men, 10 women). Participants were selected using a purposive sampling method to ensure diversity of voices, according to the following profiles: informal workers, formal workers, unemployed persons, homemakers, and self-employed persons; who lived in a marginal urban neighbourhood or were Venezuelan migrants in Bogotá.

Two instruments were used to collect information: semi-structured interviews and field diaries [24]. The interview guide (supplementary appendix 1) initially was developed by the NIHR Global Health Research Group on Oral Health (The CORE programme: https://thecoreprogramme.co.uk/). This was translated into Spanish, then culturally and linguistically adapted, and finally pilot tested. Throughout this process, consistency with the study objectives was maintained. The interview used in this research was developed for this study and not has previously been published elsewhere. The interviews lasted between 50 and 75 min, were audio-recorded, and subsequently transcribed in their entirety. The field diary allowed us to distil the experience and interaction with each interviewee, as well as our emotions and perceptions as researchers. It also facilitated the formulation of some initial interpretations, the understanding of the lived experiences and significance attributed to the mouth and oral health based on each participant’s narratives.

Data analysis followed the phenomenological parameters proposed by Marí, Bo & Climent [25]. The process began with the development of *General Meaning Units* (initial deductive/coding categorical analysis process); these led to the identification of *Relevant Meaning Units* through arrangement and grouping the data (convergences and divergences between categories); these were then triangulated across sources and face-to-face validation between researchers, which allowed for the *Verification of Units of Relevant Meaning* (final ordering by categorical groups of meaning; inductive analysis process). These Units of Relevant Meaning enabled the articulation of findings and supported the final hermeneutic-phenomenological analysis.

### Ethical considerations

The research was classified as ‘minimum risk’ [26] as it involved collecting sensitive information from participants. It adhered to the ethical principles established in UNESCO’s Universal Declaration on Bioethics and Human Rights [27], including Informed consent and Respect for cultural diversity and pluralism. Compliance with the standards of scientific integrity set out in the Singapore Statement [28] was ensured. The study was approved by the UCL Research Ethics Committee (UCL Ethics ID:1538/003) and Ethics Committee of the School of Dentistry of the Universidad Nacional de Colombia (Minutes 20–21 December 2021).

## Results

Exploring the oral health experiences of people from marginalised and vulnerable communities and Venezuelan migrants in Bogotá (Colombia) revealed that the common sense and significance they attach to the mouth and oral health in relation to care services and practices are shaped by three Units of Relevant Meaning (essential categories): *Self-confidence and social interaction*,* Barriers to accessing oral health services*,* and Bureaucratic itineraries and Dehumanisation in oral health*. These allowed us to understand that oral health is a construct both subjective and intersubjective.

### Self-confidence and social interaction

This Unit of Relevant Meaning indicates that both Colombians from marginalised and vulnerable communities and Venezuelan migrants in Bogotá share a common understanding of the significance of the mouth and oral health: suffering from oral disease and lacking adequate dental care has a substantial negative impact on personal confidence and social interaction. The social function of the mouth, normative beauty standards, impacts on self-esteem, the ideal of freedom from pain, caries and tooth loss, and access to care practices are central to this category.

#### Social function

This subcategory underscores how the mouth and oral health, through smiling, shape the way people relate to others and experience intra and inter subjective security. Smiling can facilitate social interactions, whereas its absence is often associated with —and can reinforce— feelings of shame and self-exclusion: * “when you have problems with tooth loss […] you become […] a little more complex when expressing yourself […] you can’t smile comfortably, you can’t express yourself comfortably […] you distance yourself from people, so it’s better not to talk much because it makes you feel ashamed” *(Colombian woman, 41 years old, unemployed, marginal urban neighbourhood in Bogotá).

#### Beauty standards

Participants consider a ‘beautiful’ smile and healthy teeth to be key factors in the perception of a person’s beauty and overall appearance. Social pressure to conform to beauty standards, especially from the media and social networks, often idealises a smile with straight, white teeth, directly affecting people’s lives. This standard is imposed but not equally attainable, which undermines self-esteem and confidence: *“a healthy mouth is one without cavities, without plaque, with straight teeth […] like a beauty queen” *(Man, 30 years old, informal worker, Venezuelan migrant).

**Fig. 1:**
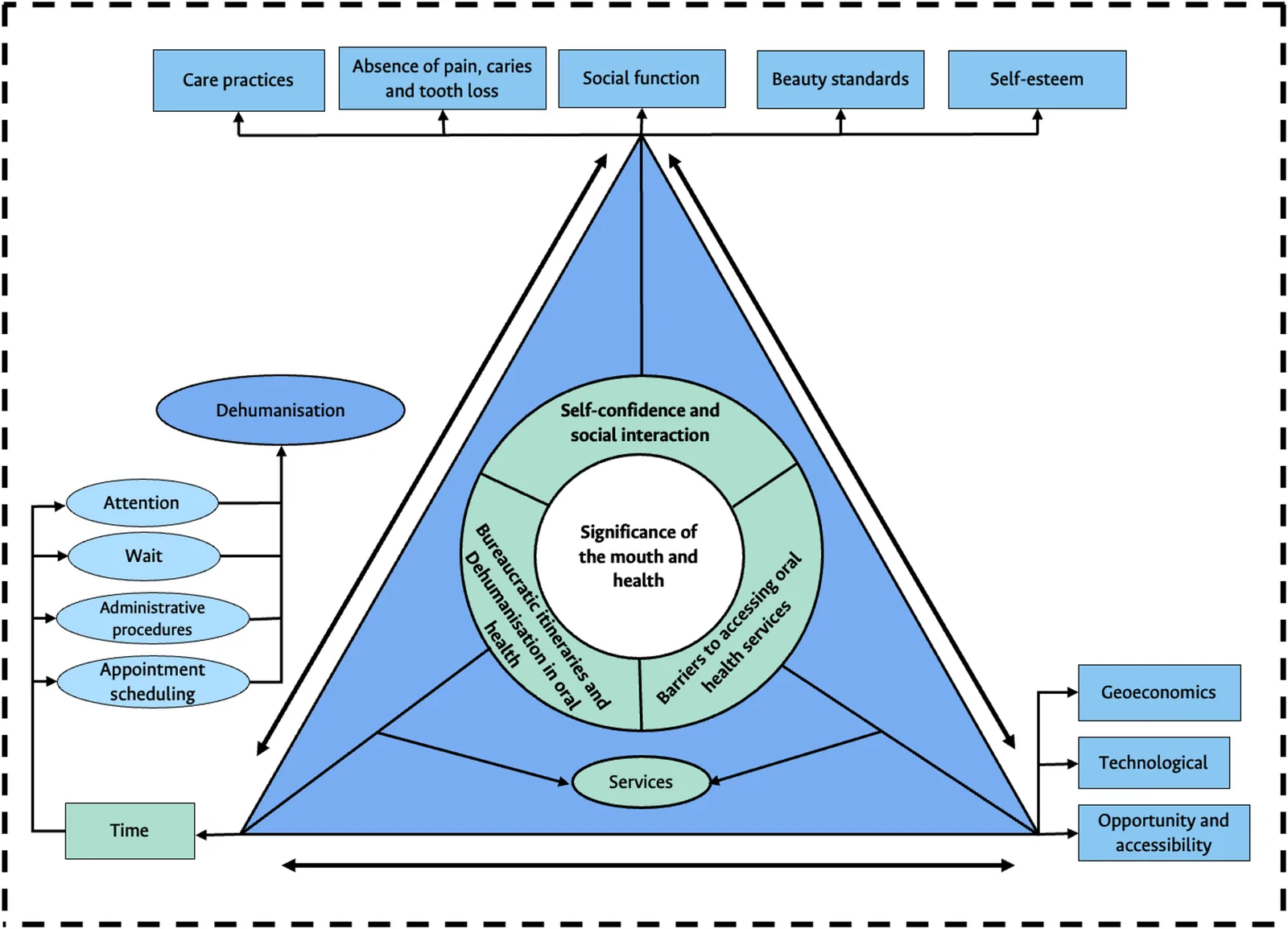
Horizon of meaning for understanding the mouth and oral health care processes

#### Self-esteem

Oral health strongly influences self‑esteem: *“For me, my teeth are the worst problem I could have had. Because first, when I didn’t have a tooth, […] they called me “broken bridge”. And obviously, that caused certain things in me, to the point that I even […] hid myself away crying”* (Man, 30 years old, informal worker, Venezuelan migrant). Tooth loss and oral diseases often markedly reduce self‑esteem, hinder social interaction, and in many cases contribute to feelings of stigmatisation and discrimination among those affected.

#### Absence of pain, caries and tooth loss

This subcategory revealed that the absence of pain, caries and tooth loss is something people long for and a fundamental condition for understanding a healthy mouth. It is related to both self-care practices and care provided by oral health professionals. Pain, caries and tooth loss cause discomfort and affect quality of life, socially and emotionally, leading people to abandon even their work roles: *“This tooth that is bothering me right now started bothering me in December… I was working as a pool lifeguard, and the toothache prevented me from working”* (Colombian man, 33 years old, informal worker, marginal urban neighbourhood in Bogotá).

#### Care practices

Interviewees recognise that brushing, flossing (as self-care practices) and regular visits to the dentist (as professional care practices) are essential for maintaining oral health and preventing problems that affect self-esteem, personal confidence, interpersonal relationships, and overall health: *“with good brushing, after every meal, not eating sweets at night and [not] going to bed without washing your mouth, using mouthwash, dental floss, […] going every six months for a cleaning, because even if you do that, they’re never 100% clean. You always need a little help with cleaning”* (Woman, 45 years old, unemployed, Venezuelan migrant). Adhering to these practices, however, is challenging for people from marginalised and vulnerable communities, due to both socioeconomic constraints and precarious access to dental care services. Figure 1 schematically shows our findings and the interaction between the different categories and subcategories.

### Barriers to accessing oral health services

The findings indicate that both groups of participants agree that access to oral health care is hampered by geo-economic, opportunity and accessibility, and technological barriers. Geo-economic barriers involve aspects related to time, place and money, which operate in combination. Opportunity- and accessibility-related barriers prevent individuals from interacting with the health system in Colombia, leaving behind those who do not fit within the parameters pre-established by institutions or by various mechanisms within the system. Technological barriers represent a newer obstacle, arising from the lack of accessibility and inclusivity, especially for certain human diversities and for people without formal or digital literacy [29].

#### Geo-economics

Most participants, particularly the unemployed and informal workers, faced difficulties accessing public healthcare services (hospitals and dental centres) due to the distance between these facilities and their homes. This distance implied additional expenses and, in many cases, a loss of income from the suspension of work activities: “*It takes* *an* *hour and a half*,* almost two hours on* *Transmilenio* *(bus transport system)* *to get there. […] And there are other options nearby, as there are more [of that entity’s] locations, but the one where they treat me is there”* (Colombian woman, 36 years old, housewife, marginal urban neighbourhood in Bogotá). It was evident that better economic conditions meant greater ease of access: *“The barriers are more economic than anything else. Because I have heard from other co-workers that those who belong to the same EPS (health insurer) but are under the contributory regime do receive prompt care. On the other hand, those who are subsidised (health insurance scheme), whether they are Venezuelan, Colombian, or from wherever, if they are subsidised, no!”* (Man, 35 years old, self-employed, Venezuelan migrant).

#### Opportunity and accessibility

Participants faced long waiting times to access appointments and had to navigate bureaucratic procedures before receiving care. This situation hindered access to timely and adequate care, leading those with financial resources to seek alternatives in the private sector: *“I called to schedule an appointment to have my tooth extracted […] so I wouldn’t have to pay privately, but there were never any appointments […]. You have to call, you have to wait, so I’ve been in that process for a month… I kept calling to have it removed, but so far I haven’t been able to get an appointment, so I had to go privately”* (Colombian woman, 36 years old, housewife, marginal urban neighbourhood in Bogotá). For Venezuelan migrants, the lack of legal documents was an obstacle that created barriers of opportunity and accessibility, as it prevented them from interacting with the healthcare system in Colombia. Administrative procedures were perceived as an endless chain of steps that consumed time, energy, and both financial and vital resources. The absence of documentation or the slowness in obtaining it rendered them socially invisible and unable to access basic rights: *“They don’t treat them because they don’t have the PPT document (Venezuelans foreigners ID in Colombia). […] They are automatically denied care in the health sector”* (Woman, 36 years old, informal worker, Venezuelan migrant). Those without financial resources were forced to live with the pain or to rely on temporary solutions, which often led to accepting permanent consequences such as tooth extraction.

#### Technological

The method of scheduling appointments by telephone often results in a frustrating experience, as calls are not answered, waiting times are long, switchboard options are confusing, or appointments are not available: *“It’s almost impossible to schedule an appointment because, first of all, they almost never answer the phone […] to request an appointment directly. So, we’ve practically given up on that […], the website doesn’t work either, so you’re left hanging”* (Male, 35 years old, self-employed, Venezuelan migrant). In addition, the lack of inclusive design in digital tools excludes people with disabilities or those who are digitally illiterate (some elderly adults, among others), turning technology into a new obstacle: *“websites are not normally accessible […] the machines that give appointments… are touch-sensitive and are not adapted either, […] I’m going to press all the buttons because I can’t find the one I need”* (Colombian man, 28 years old, formal worker, marginal urban neighbourhood in Bogotá).

### Bureaucratic itineraries and dehumanisation in oral health

This Unit of Relevant Meaning indicates that bureaucratic itineraries [30, 31] in accessing oral healthcare are a common reality for Colombians from marginalised and vulnerable communities and for Venezuelan migrants in Bogotá. Participants generally perceived the Colombian healthcare system as neglected and hostile, shaped by complex and dehumanising procedures that consume time, increase suffering, and negatively affect their lives. Patients reported feeling treated as objects rather than as human beings. After overcoming multiple obstacles to access services, even their bodies bore visible marks of inequality, including tooth loss.

#### Time

Given the considerable amount of time participants spent on bureaucratic itineraries—from requesting an appointment to receiving treatment —they often felt discouraged from seeking oral health care and reported negative repercussions at work:* “When it came to taking time off work […] I was also very reluctant to request appointments for many things, because I missed several appointments at work […] And I missed like two or three appointments because my boss said. No, we’re too busy, you can’t” *(Woman, 45 years old, unemployed, Venezuelan migrant). In addition, the time allocated to the consultation itself was perceived as inadequate and inefficient. Participants noted that the 15–20 min of the appointment were often insufficient, as much of that time was spent on administrative procedures, leaving little room for attending to and caring for their oral health problems: *“The delay in appointments more than anything else […] I arrived today at 6:40 in the morning, and they gave me ticket number 49. I didn’t get to the window until 7:40 […] I wasn’t seen until 9:20”* (Colombian woman, 58 years old, informal worker, marginal urban neighbourhood in Bogotá).

#### Dehumanisation

Dehumanisation emerged as a corollary closely linked to the two previous categories and the bureaucratic itineraries. Participants felt treated more like objects than human beings —undignified treatment— describing interactions that reflected a lack of interest in their well-being and quality of life, both from the system and from professionals. This experience was particularly acute for Venezuelan migrants facing prejudice and discrimination, and for people with functional diversity: *“Be truly aware that you are dealing with a human being, not an animal… you have to be a little more humanised when dealing with another person” *(Colombian man, 28 years old, formal worker, marginal urban neighbourhood in Bogotá).

## Discussion

This research showed that oral health has a significant impact on people’s self-confidence and social interaction. Three types of barriers to accessing oral health services were identified: geo-economic, opportunity‑ and accessibility‑related, and technological. A marked difference was observed between the subsidised and contributory schemes, with the latter perceiving fewer access barriers. In addition, a process of dehumanisation in care was evident, reflected in undignified treatment and bureaucratic itineraries that consume time and demotivate patients.

In relation to personal confidence and social interaction, participants emphasised that the social function of the mouth is fundamental to social relationships and self-perception. The fear of being judged by one’s ‘dental appearance’ can limit social participation and affect self-esteem, in both women and men. These findings confirm those of Militi et al. [32]. who found a positive and significant correlation (r = + 0.62) between concern for dental aesthetics and psychosocial impact. This implies that the greater the aesthetic concern, the greater the negative impact on psychological well-being and social interaction. Studies such as that by Taghavi et al. [33] complement these results, pointing out that the influence of the media reinforces low self-esteem by promoting unattainable aesthetic ideals, leading to feelings of insecurity and the adoption of behaviours such as avoiding smiling, self-isolation, or covering mouth, with possible negative consequences for social life. In this context, it is evident that society establishes standards of beauty that many people, especially those who are marginalised and vulnerable, find difficult or impossible to achieve, contributing to their insecurity and perception of rejection, as expressed by both our Colombian and Venezuelan participants.

Another key element in the perception of adequate personal confidence and social interaction is the absence of pain, caries, and tooth loss, something that people long for and a fundamental condition in understanding a healthy mouth. These elements are the antithesis of well-being and quality of life from an oral health perspective, impacting not only physical health but also the ability to concentrate, work and participate actively in all aspects of social life. Although there are no qualitative studies related to these categories, our results coincide with Salvador and Toassi [34], who identified that the presence of pain can limit essential daily activities such as eating, affect concentration and social interactions, and impact work productivity.

This study also revealed that geo‑economic barriers restrict access to oral health services, with financial constraints, long displacement, and the location of health centres being the main contributing factors. Most participants affiliated with the subsidised scheme indicated that out-of-pocket costs, long distances, and travel times prevent them from receiving timely care. These results reinforce the findings of Caicedo–Rosero and Estrada–Montoya [17] and Estrada–Montoya and Reyes [18], who found that travel time and means of transport act as barriers to access, especially in rural and hard-to-reach areas in Colombia. Our results show that barriers to accessing oral health services in Colombia are not exclusive to rural areas, and that they involve a combination of geo-economic, technological and structural, which also disproportionately affect marginalised and vulnerable populations living in urban areas.

The participants’ experiences show that barriers related to availability and accessibility, together with the lack of prioritisation in cases of pain or emergencies, lead to a situation where more affluent people turn to the private sector, while those without sufficient financial means are forced to endure the pain, live with the condition, and ultimately undergo tooth extraction when possible. This situation is consistent with the findings of Teixeira et al. [35]. who observed that adverse socio‑economic conditions throughout life are determinants of dental care, making the mouth an indicator of social inequality. Tooth loss can therefore be understood as an embodied inequality, as suggested by Silberman et al. [36]. By often defaulting to tooth extraction as a response to problems that could be prevented or treated in other ways, the Colombian health system reinforces these inequalities. For the Venezuelan migrant participants in this study, the absence of legal documents represents an initial barrier that excludes them from the health system. This is consistent with the findings of Hacker et al. [37], and Chandler et al. [38]. who identified this as a main barrier for undocumented migrants and their families in various countries.

Regarding technology, it was found that its use in the healthcare sector in general, and in oral health in particular, is limited by factors such as inadequate digital infrastructure, an ageing population, and digital illiteracy. Pacheco–Gallego and Rubiano–Pavía [29] point out that, although technological tools are designed to improve processes and facilitate actions, they actually represent a barrier for groups that lack digital skills, face generational gaps, or distrust technology, as we have seen in this study. These barriers can lead to digital exclusion, delay treatment, and limit opportunities. Inadequate digital infrastructure could particularly affect people with disabilities, as many platforms and devices do not incorporate the necessary commands or adaptations [39]. It is therefore a priority to promote inclusive platforms with adapted commands and compatibility with assistive technologies, and promote access to these technologies in remote areas given their potential to improve oral health outcomes by expanding access to teledentistry and certain prevention strategies in such settings [40]. For future research, it would be valuable to explore and understand how technologies and technological barriers to access may contribute to accentuating inequalities in access to health services.

Regarding bureaucratic itineraries in oral health, lengthy administrative processes and complex documentation requirements delay care, aggravate oral health, and favour the perpetuation of entrenched inequalities. These findings coincide with those of Abadía and Oviedo [30, 31], who argue that bureaucratic itineraries depend more on compliance with administrative rules and costs than on people’s needs. These itineraries generate negligence, inefficiencies, and inequalities, and can lead to permanent damage, increased pain, and the need for radical procedures such as tooth extraction, with detrimental consequences for people’s lives. In the same vein, the findings of Murillo‑Pedrozo et al. [41] showed that immigration status was the main factor determining access to healthcare services, followed by economic factors; these are compounded by the multiple procedures and bureaucratic itineraries that patients must navigate to receive medical care and, in many cases, ultimately face the denial of their right to healthcare.

Dehumanisation in dental care processes highlights the profound depersonalisation experienced by patients in certain institutional contexts and the perception of undignified treatment. In contrast, dignified and humanised treatment could result in a better perception of the healthcare system, especially because it addresses patients’ needs and promotes quality care. Authors such as Oliveira et al. [42], Oliveira et al. [43], and Camps [44, 45] agree with our reflection and findings, arguing that humanisation during oral health care processes is a challenge for both institutions and dentists. According to our participants, humanisation is related to comprehensive care that combines the technical and scientific aspects of dentistry with elements of assertive and intersubjective communication, mutual recognition and dentist-patient empathy, resulting in dignified treatment.

Dignified treatment must be mediated by the practice of ethical virtues such as benevolence, compassion, respect, sincerity, kindness, and prudence, among others; bioethical principles such as respect for cultural diversity and pluralism, respect for human vulnerability and personal integrity, non-discrimination and non-stigmatisation, and human dignity and human rights, among others; and to cement in institutions and dentists a perspective of hospitality and ethics of care. For its part, the Colombian Health System will have to be designed with respect for patient dignity and a person-centred approach during oral health care and treatment processes. The Colombian health system must therefore recognise the human right to oral health and the removal of barriers to access associated with opportunity and accessibility as matters of bioethics and social justice, and in this way seek to abolish the so-called “inverse care law”.

Among the strengths of this study was the inclusion of two unheard populations: Venezuelan migrants and communities in marginalised neighbourhoods in Bogotá. The study provided an opportunity to express their experiences in relation to oral health care, and numerous people participated. In terms of limitations of study, the possibility of social desirability bias is acknowledged. However, this bias was mitigated, as none of the interviewees knew the researchers, which encouraged more honest and authentic responses.

## Conclusions

Based on the voices of these marginalised and vulnerable populations, it can be concluded that the mouth and oral health are fundamental to people’s lives; not only do they influence people’s perception of themselves and their self-esteem, but they also affect their relationships with others, in terms of intersubjectivity, in public or social life. In addition to the traditional barriers identified in the literature (opportunity‑ and accessibility‑related), it is concluded that geoeconomic and technological barriers are present in oral health care processes, which are accentuated by the presence of bureaucratic procedures, negatively impacting people’s quality of life and well-being. Therefore, dental care services and public policies on oral health must be rethought to effectively ensure more equitable access for marginalised and vulnerable populations. This study lays the groundwork for future research and opens the possibility of exploring mixed-methods designs and involving other relevant actors in the field of oral health care, thereby enriching knowledge and interventions in this area.

## Supplementary Information

Below is the link to the electronic supplementary material.

Supplementary Material 1

Supplementary Material 2

## Data Availability

The datasets generated and/or analysed during the current study are not publicly available due ethical considerations but are available from the corresponding author on reasonable request.
