# Supplementary material for: Unheard voices in oral health: the mouth, oral health, and barriers to accessing dental care in disadvantaged communities and Venezuelan migrants in Bogotá
Source: Int J Equity Health. 2026 Jul 17;25:242. doi: 10.1186/s12939-026-02925-9 (PMC13663294; doi:10.1186/s12939-026-02925-9)
Supplement: Supplementary file 1 [file 12939_2026_2925_MOESM2_ESM.docx]

**Topic Guide: Interviews and Group Discussions**

***Introduction and overview of study and steps involved***

*Thanks for agreeing to be involved in this study.*

*Personal introductions from research staff.*

*Recap on aim of study and general details.*

*Recap on purpose and details of the interview/discussion – last approx. 45 min.*

*Stress confidentiality of process – no names or personally identifiable information to be reported.*

*Stress right to withdraw at any point.*

*Ask permission to audio/video record.*

*Ask if questions and if okay to proceed.*

**Points for discussion**

*A: General opening and ‘warm-up’ - introductions*

**1. Tell me a little bit about yourself?**

**2. How long have you lived in this area- what’s it like around here?**

**3. How would you describe your health – are you well?**

*B: Exploration of personal experience of oral diseases and their impact.*

**4. I would like to learn more about your oral health – are your teeth, gums and mouth healthy?**

**5. Can you describe what you think are signs of a healthy mouth?**

**6. How important are your teeth and mouth?**

**7. Have you had any particular problems with your teeth and mouth at any point in your life (including childhood)?**

**8. Tell me more about these problems – how did they affect you?**

*C: Exploring views on the causes of oral diseases and opportunities for prevention*

**9. What do you think causes problems with people’s teeth and mouth?**

**10. Can anything be done to stop these problems from happening?**

**11. What advice would you give your family or friends for them to look after their teeth and mouth?**

*D: Exploration of personal experience of using local dental services and barriers to access*

**12. Have you ever been to a dentist? If yes, tell me more about your experience of visiting a dentist (include recent visits and memorable visits if any)?**

**13. More generally, are dentists available in this area and what do you think of them as providers of dental care?**

**14. Apart from dentists, does anyone else around here help people if they have problems with their teeth or mouth?**

**15. What are the main barriers stopping local people from seeing a dentist in this area?**

*E: Exploration of views on how to improve local dental services and how to involve local people in developing future dental services.*

**16. How can local dental services in this area be improved?**

**17. If people like you had problems with their teeth and mouth, how could they be helped in this area?**

**18. How could local people like you get involved in improving local dental services?**

*F: Closing remarks.*

**Thanks for your time and very useful comments. Check if missed anything important. Recap on confidentiality and explain next steps. Provide oral health information sheet and/or general advice.**
